# Construction of a risk prediction model for postoperative deep vein thrombosis in colorectal cancer patients based on machine learning algorithms

**DOI:** 10.3389/fonc.2024.1499794

**Published:** 2024-11-27

**Authors:** Xin Liu, Xingming Shu, Yejiang Zhou, Yifan Jiang

**Affiliations:** ^1^ Department of Clinical Medicine, Southwest Medical University, Luzhou, China; ^2^ Department of Gastrointestinal Surgery, The Affiliated Hospital of Southwest Medical University, Luzhou, Sichuan, China

**Keywords:** colorectal cancer, venous thrombosis, machine learning, prediction model, postoperative complications

## Abstract

**Background:**

Colorectal cancer is a prevalent malignancy of the digestive system, with an increasing incidence. Lower extremity deep vein thrombosis (DVT) is a frequent postoperative complication, occurring in up to 40% of cases.

**Objective:**

This research aims to develop and validate a machine learning model (ML) to predict the risk of lower limb deep vein thrombosis in patients with colorectal cancer, facilitating preventive and therapeutic measures to enhance recovery and ensure safety.

**Methods:**

In this retrospective cohort study, we collected data from 429 colorectal cancer patients from January 2021 to January 2024. The medical records included age, blood test results, body mass index, underlying diseases, clinical staging, histological typing, surgical methods, and postoperative complications. We employed the Synthetic Minority Oversampling Technique to address imbalanced data and split the dataset into training and validation sets in a 7:3 ratio. Feature selection was performed using Random Forest (RF), XGBoost, and Least Absolute Shrinkage and Selection Operator algorithms (LASSO). We then trained six machine learning models: Logistic Regression (LR), Naive Bayes (NB), Gaussian Process (GP), Random Forest, XGBoost, and Multilayer Perceptron (MLP). The model’s performance was evaluated using metrics such as area under the Receiver Operating Characteristic curve, accuracy, sensitivity, specificity, F1 score, and confusion matrix. Additionally, SHAP and LIME were used to enhance the interpretability of the results.

**Results:**

The study combined Random Forest, XGBoost algorithms, and LASSO regression with univariate regression analysis to identify significant predictive factors, including age, preoperative prealbumin, preoperative albumin, preoperative hemoglobin, operation time, PIKVA2, CEA, and preoperative neutrophil count. The XGBoost model outperformed other ML algorithms, achieving an AUC of 0.996, an accuracy of 0.9636, a specificity of 0.9778, and an F1 score of 0.9576. Moreover, the SHAP method identified age and preoperative prealbumin as the primary determinants influencing ML model predictions. Finally, the study employed LIME for more precise prediction and interpretation of individual predictions.

**Conclusion:**

The machine learning algorithms effectively predicted postoperative lower limb deep vein thrombosis in colorectal cancer patients. The XGBoost model demonstrated strong potential for improving early detection and treatment in clinical settings.

## 1 Introduction

Colorectal cancer is among the most prevalent malignant tumors of the digestive system globally, ranking third in both incidence and mortality rates among malignant tumors (1). Currently, surgical treatment is the primary approach for colorectal cancer. However, ostoperative lower limb deep vein thrombosis has consistently been an issue that cannot be overlooked. Literature reports that the incidence of lower limb deep vein thrombosis after abdominal surgery is 15%-19%. Alarmingly, the incidence in colorectal cancer patients post-surgery is 40% (2). Additionally, since only 50% of patients with lower limb deep vein thrombosis exhibit symptoms and signs such as swelling and tenderness, many cases are overlooked postoperatively (3). Without timely diagnosis and intervention, the clot may detach and move through the veins to the lungs, leading to a life-threatening pulmonary embolism (4). However, lower limb deep vein thrombosis can be prevented in advance. Research suggests that prophylactic anticoagulant treatment can be suitably applied to bedridden patients in the perioperative phase (5, 6). Currently, the Caprini risk assessment model is the most widely used model in surgery. However, all colorectal cancer patients stratified postoperatively according to the Caprini model are considered high risk. Therefore, the Caprini model may not be a completely accurate indicator for DVT occurrence and intervention in colorectal cancer patients (7).

Additionally, most existing studies utilize traditional statistical methods rather than advanced machine learning algorithms, which often limits the models’ ability to handle nonlinear relationships and multivariable interactions, thereby affecting their predictive performance and applicability (8). The purpose of this study is to integrate these common high-risk factors using machine learning by selecting shared features through three different machine learning algorithms and constructing multiple models to identify the optimal deep vein thrombosis risk prediction model for colorectal cancer patients. This model will assist clinicians in more accurately identifying high-risk patients and providing personalized, precise guidance for the prevention and treatment of deep vein thrombosis.

## 2 Materials and methods

### 2.1 Study design

**Figure 1:**
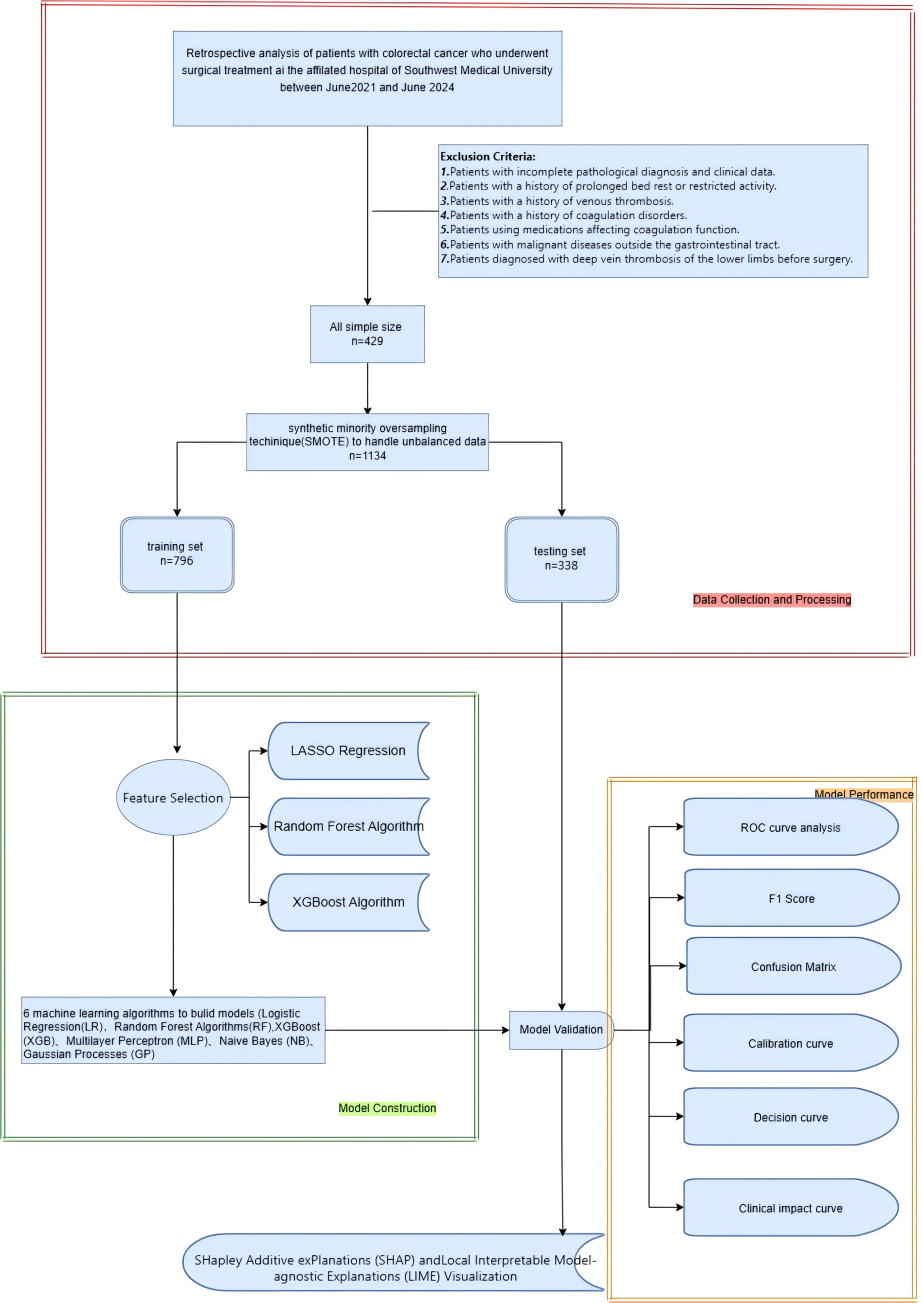
Research process.

The aim of this research is to develop a machine learning-based model to predict the risk of lower limb deep vein thrombosis in postoperative colorectal cancer patients. A retrospective study was conducted, including 429 colorectal cancer patients who underwent surgical treatment. Data were extracted from the hospital’s electronic medical record system, which included demographic details, medical history, treatment information, disease severity, blood test results, and postoperative complications. The SMOTE algorithm was employed to address the issue of class imbalance. LASSO regression, Xgboost, and random forest were applied for feature selection to identify the features most associated with the risk of lower limb deep vein thrombosis. Following this, a range of ML models, such as LR, RF, GB, MLP, XGB, and KNN, were developed and optimized using the 10-fold cross-validation approach. The performance of these models was assessed through a range of metrics, including accuracy, sensitivity, specificity, positive predictive value, negative predictive value, F1 score, Kappa score, AUC, calibration curve, clinical impact curve, and confusion matrix. To enhance the transparency and interpretability of the model, SHAP and LIME methods were used to explain the prediction results, clarifying the impact of each feature on the predictions and thereby offering useful references for clinicians. **Figure 1** illustrates the overall workflow of the proposed system more clearly.

### 2.2 Study data

We retrospectively selected 429 colorectal cancer patients who visited the Department of Gastrointestinal Surgery at the First Affiliated Hospital of Southwest Medical University from January 2022 to January 2024. Exclusion criteria include: patients with a history of prolonged bed rest or restricted activity; patients with a history of venous thrombosis; patients with a history of coagulation disorders; patients using drugs affecting coagulation function; patients with malignancies outside the gastrointestinal tract; and patients preoperatively diagnosed with lower extremity deep vein thrombosis. (Exclusion criteria are shown in **Figure 1**). As this study is retrospective, patients are exempt from providing informed consent according to the ethics review board’s policy. The ethics committee has encrypted all personal information of patients involved in this study to prevent any leaks.

### 2.3 Study variables

The study includes 44 variables related to demographic factors (gender, age), medical history (history of diabetes, hypertension, coronary artery disease, chronic obstructive pulmonary disease), physical characteristics (BMI), disease severity (clinical stage, histological grade, presence of cancer embolus, nerve invasion, vascular invasion), treatment information (surgical method, surgery duration, use of specific cancer treatments), laboratory values (white blood cell count, neutrophil count, lymphocyte count, monocyte count, NLR, hemoglobin, prealbumin, albumin, creatinine clearance, platelet count, prothrombin time PT, fibrinogen, thrombin time TT, D-dimer), and postoperative complications (postoperative high fever, anastomotic leak). Venous blood samples were collected within 24 hours of admission.

### 2.4 Diagnosis

Patients were tested within 14 days postoperatively according to the diagnostic criteria for lower limb deep vein thrombosis. Specifically, color Doppler ultrasound showed an uneven echo solid mass in the lower limb, reduced or absent color blood flow and spectral signals, non-collapse of the venous lumen after compression, and venous incompressibility (9).

### 2.5 Data preprocessing

The structured database initially included 44 clinical variables. First, clinical variables with more than 30% missing data (n = 2) were excluded. The missing data were handled using 10-fold crossvalidation combined with the KNN imputation method. Subsequently, to prevent bias during later model training and improve interpretability, the Variance Inflation Factor (VIF) was employed to examine multicollinearity among the chosen features, ensuring all features’ VIF values were less than 10. Additionally, we also removed variables with nearly zero variance to simplify the model and enhance its robustness. In the end, 39 clinical features of patients were chosen to construct the predictive model. The SMOTE algorithm was used to address the class imbalance issue, balancing the dataset and avoiding bias. Subsequently, patient data were randomly divided into two datasets: (1) a training dataset (70%) for feature selection and model training, and (2) a testing dataset (30%) for model performance evaluation.

### 2.6 Feature selection

For predicting postoperative DVT occurrence in colorectal cancer patients, features were selected using training group samples through three machine learning models: LASSO regression, random forest, and XGboost. The results showed that 29, 15, and 15 feature vectors were selected in the three models, Ultimately, we selected 8 common feature variables from the three models: age, preoperative prealbumin, preoperative albumin, preoperative hemoglobin, CEA, PIKVA2, surgery time, and preoperative white blood cell count.

### 2.7 Model development and evaluation

The machine learning task is to predict the probability distribution of patients developing lower extremity deep vein thrombosis based on these clinical variables. Model development involves experimenting with six machine learning algorithms: Logistic Regression (LR), Multilayer Perceptron (MLP), Extreme Gradient Boosting (XGBoost), Gaussian Process (GP), Random Forest (RF), and Naive Bayes (NB). During the training phase, we employed the 10-fold cross-validation method to train the models in order to achieve optimal predictive performance. To evaluate the predictive performance of each model, we primarily measured the Receiver Operating Characteristic (ROC) curve. In addition, we calculated sensitivity, specificity, accuracy, false positive (FP) rate, positive predictive value (PPV), negative predictive value (NPV), Brier score, F1 score, Decision Curve Analysis (DCA) curve, calibration curve, and Clinical Impact Curve (CIC) for a comprehensive assessment of the model’s performance.

### 2.8 Statistical analysis

All data analyses in this study were carried out using SPSS (27.0) and R language (version 4.3.3). Preliminary analysis of the dataset used descriptive statistics. Data points that followed a normal distribution were represented by mean ± standard deviation, whereas data points deviating from a normal distribution were shown as median (interquartile range). Subsequently, an independent samples t-test was employed to compare two groups of normally distributed data. In contrast, the Mann-Whitney U test was used for comparing two groups of non-normally distributed data. We resolved the sample imbalance problem by oversampling the minority classes using the SMOTE function from the DMwR2 package in R. To build the predictive model, the dataset was randomly split into a training subset comprising 70% of the total data and a testing subset making up 30% of the total data. Subsequently, various machine learning methods were executed using R, including logistic regression (glm package), Gaussian model (e1071 package), random forest (randomForest package), XGBoost (XGBoost package), feedforward neural network (nnet package), and naive Bayes model (e1071 package). Models were trained using the training subset data with these six ML algorithms. During the model training, a 10-fold cross-validation method was adopted to optimize the model parameters, aiming to prevent overfitting. Statistical significance was defined at the level of P<0.05.

### 2.9 Feature interpretation

We used the Shapley Additive Explanations (SHAP) algorithm and the Local Interpretable ModelAgnostic Explanations (LIME) algorithm to interpret the main feature contributions after machine learning model training. In particular, the SHAP algorithm assesses the average contribution of each feature value by computing its Shapley value within all possible combinations of features. By taking the weighted average of each feature value’s Shapley value, we can assess the impact of that feature on the overall prediction. Meanwhile, the LIME algorithm analyzes the model from a local perspective to explain the feature importance of specific predictions, providing an additional layer of interpretation and transparency. The combination of these two methods provides us with a multidimensional understanding of model interpretability.

## 3 Results

### 3.1 Characteristics of patients

This study encompassed 429 colorectal cancer patients who underwent surgical treatment. The median age of the patients was 67 years (range: 16-91), with 258 males (60.24%) and 171 females (39.76%). The original data from 429 cases includes 267 cases without lower extremity deep vein thrombosis (62.23%) and 162 cases with lower extremity deep vein thrombosis (37.77%). The baseline characteristics comparison of the two patient groups in the original data reveals that age, preoperative white blood cell count, preoperative lymphocyte count, preoperative hemoglobin, preoperative albumin, preoperative prealbumin count, preoperative glomerular filtration rate, gender, preoperative acute complete intestinal obstruction, and surgical method are all statistically significant (refer to **Table 1**).

**Table 1:** Raw data in Three-Baseline table.

Variables	Total (n=429)	Missing Value (%)	Deep Vein Thrombosis Occurrence After Colorectal Surgery		P
			N0 (n=267)	YES (n=162)	
age	67 (57-73)	0	61 (54-71)	71 (65-74)	<0.01
prewbc	6.57 (5.25-8.08)	0	6.51 (5.34-7.58)	6.72 (5.12-8.89)	<0.01
prene	4.31 (3.27-5.77)	0	4.27 (3.28-5.35)	4.44 (3.26-6.38)	<0.01
prelym	1.37 (1.07-1.75)	0.7	1.39 (1.12-1.79)	1.32 (1.02-1.72)	0.06
premon	0.39 (0.3-0.51)	0.7	0.37 (0.3-0.5)	0.4 (0.31-0.53)	0.48
preNLR	68.3 (61.2-75.08)	0	67.4 (60.1-73.7)	70.25 (63.25-76.95)	0.08
prehb	126 (111-140)	0	131 (115-143)	120 (103-129)	<0.01
prepab	194.9 (156.3-230.2)	0.2	207.3 (176.32-242.95)	165.3(136.12-201.9)	<0.01
prealb	41.5 (38.6-44.1)	0	42.3 (39.95-44.9)	39.75 (37.23-42.5)	<0.01
precrci	93.05 (82.77-102.2)	0.2	95.9 (84.65-105.35)	90.8 (80.9-98)	<0.01
preplt	235 (194.75-304)	0.2	230 (191.5-299.75)	246 (198.25-318)	0.1
AFP	2.93 (2.14-3.96)	1.4	2.99 (2.21-4.08)	2.82 (2.02-3.66)	0.12
CEA	5.64 (3.08-15.25)	1.2	5.25 (3.07-13.88)	6.05 (3.18-19.52)	0.08
FER	64.18 (21.79129.28)	2.6	74.44 (26.35128.98)	45.53 (15.5-130.88)	0.91
CA50	8.92 (4.54-18.37)	2.8	8.92 (4.19-15.6)	8.91 (4.83-23.03)	0.13
CA242	5.66 (2.9-14.61)	2.8	5.66 (3.05-13.57)	5.67 (2.63-18.53)	0.3
CA724	2.83 (1.29-7.97)	2.8	2.82 (1.27-8.13)	2.96 (1.38-6.49)	0.46
CA199	12.03 (3.91-24.02)	2.1	11.5 (3.78-21.81)	14.68 (4.34-26.86)	0.03
PIVKA2	23.69 (18.37-31.35)	2.6	24.52 (18.72-31.68)	22.99 (18.26-30.52)	0.46
prept	11.3 (10.9-12)	1.4	11.3 (10.9-11.9)	11.4 (11-12.12)	0.14
prefib	3.69 (3.02-4.27)	1.4	3.58 (3-4.16)	3.83 (3.13-4.49)	<0.01
prett	17 (16.15-17.8)	1.4	17.1 (16.3-17.8)	16.8 (16.1-17.6)	0.08
pred2	0.4 (0.3-0.6)	53.4	0.37 (0.27-0.55)	0.46 (0.33-0.8)	0.95
time	225 (195-265)	0.7	220 (185.5-260)	234 (200-270)	0.01
Blood	50 (20-50)	4	50 (20-50)	50 (20-50)	0.08
BMI	22.77 (20.72-24.97)	4.9	22.77 (20.96-24.84)	22.83 (20.4-25.11)	0.94
Gender					< 0.01
Female	171 (39.86%)	0	86 (32.21%)	85 (52.47%)	
Male	258 (60.14%)		181 (67.79%)	77 (47.53%)	
Region					0.04
Ascending Colon	85 (19.81%)	0	47 (17.60%)	38 (23.46%)	
Transverse Colon	28 (6.53%)		11 (4.12%)	17 (10.49%)	
Descending and Sigmoid Colon	89 (20.75%)		60 (22.47%)	29 (17.90%)	
Upper-middle Rectum	154 (35.90%)		101 (37.83%)	53 (32.72%)	
Lower rectum	73 (17.02%)		48 (17.98%)	25 (15.43%)	
Obstruction					<0.01
No	379 (88.34%)	0	248 (92.88%)	131 (80.86%)	
Yes	50 (11.66%)		19 (7.12%)	31 (19.14%)	
Specialreatment					1
No	312 (72.73%)	0	194 (72.66%)	118 (72.84%)	
Yes	117 (27.27%)		73 (27.34%)	44 (27.16%)	
stag					0.1
Stag0	11 (2.56%)	0	9 (3.37%)	2 (1.23%)	
StagI	30 (6.99%)		22 (8.24%)	8 (4.94%)	
StagII	174 (40.56%)		110 (41.20%)	64 (39.51%)	
StagIII	155 (36.13%)		97 (36.33%)	58 (35.80%)	
StagIV	59 (13.75%)		29 (10.86%)	30 (18.52%)	
tissue					0.82
Intramucosal Carcinoma	6 (1.40%)	0.2	5 (1.88%)	1 (0.62%)	
Highly Differentiated Adenocarcinoma	46 (10.75%)		29 (10.90%)	17 (10.49%)	
Moderately Differentiated Adenocarcinoma	293 (68.46%)		183 (68.80%)	110 (67.90%)	
Poorly Differentiated Adenocarcinoma	24 (5.61%)		14 (5.26%)	10 (6.17%)	
undifferentiated carcinoma	59 (13.79%)		35 (13.16%)	24 (14.81%)	
Tumor Embolus					0.71
No	349 (81.92%)	0.7	216 (81.20%)	133 (83.12%)	
Yes	77 (18.08%)		50 (18.80%)	27 (16.88%)	
Vascular Invasion					0.05
No	323 (75.64%)	0.5	211 (79.03%)	112 (70.00%)	
Yes	104 (24.36%)		56 (20.97%)	48 (30.00%)	
Perineural Invasion					0.76
No	325 (76.11%)	0.5	205 (76.78%)	120 (75.00%)	
Yes	102 (23.89%)		62 (23.22%)	40 (25.00%)	
Microsatellites					0.16
Stable	244 (93.13%)	38.9	156 (95.12%)	88 (89.80%)	
Unstable	18 (6.87%)		8 (4.88%)	10 (10.20%)	
Hypertension					0.23
No	322 (75.41%)	0.5	207 (77.53%)	115 (71.88%)	
Yes	105 (24.59%)		60 (22.47%)	45 (28.12%)	
Diabetes					0.31
No	372 (87.32%)	0.7	237 (88.76%)	135 (84.91%)	
Yes	54 (12.68%)		30 (11.24%)	24 (15.09%)	
CAD					0.39
No	402 (94.59%)	0.9	255 (95.51%)	147 (93.04%)	
Yes	23 (5.41%)		12 (4.49%)	11 (6.96%)	
Pneumonia					0.09
No	387 (90.85%)	0.7	248 (92.88%)	139 (87.42%)	
Yes	39 (9.15%)		19 (7.12%)	20 (12.58%)	
Approach					<0.01
Laparotomy	78 (18.22%)	0.2	36 (13.48%)	42 (26.09%)	
Laparoscopic Surgery	350 (81.78%)		231 (86.52%)	119 (73.91%)	
Fever					0.87
No	336 (78.50%)	0.2	210 (78.95%)	126 (77.78%)	
Yes	92 (21.50%)		56 (21.05%)	36 (22.22%)	
Leak					1
No	290 (97.97)	31	180 (97.83)	110 (98.21)	
Yes	6 (2.03)		4 (2.17)	2 (1.79)	

### 3.2 Prediction factor screening

**Figure 2:**
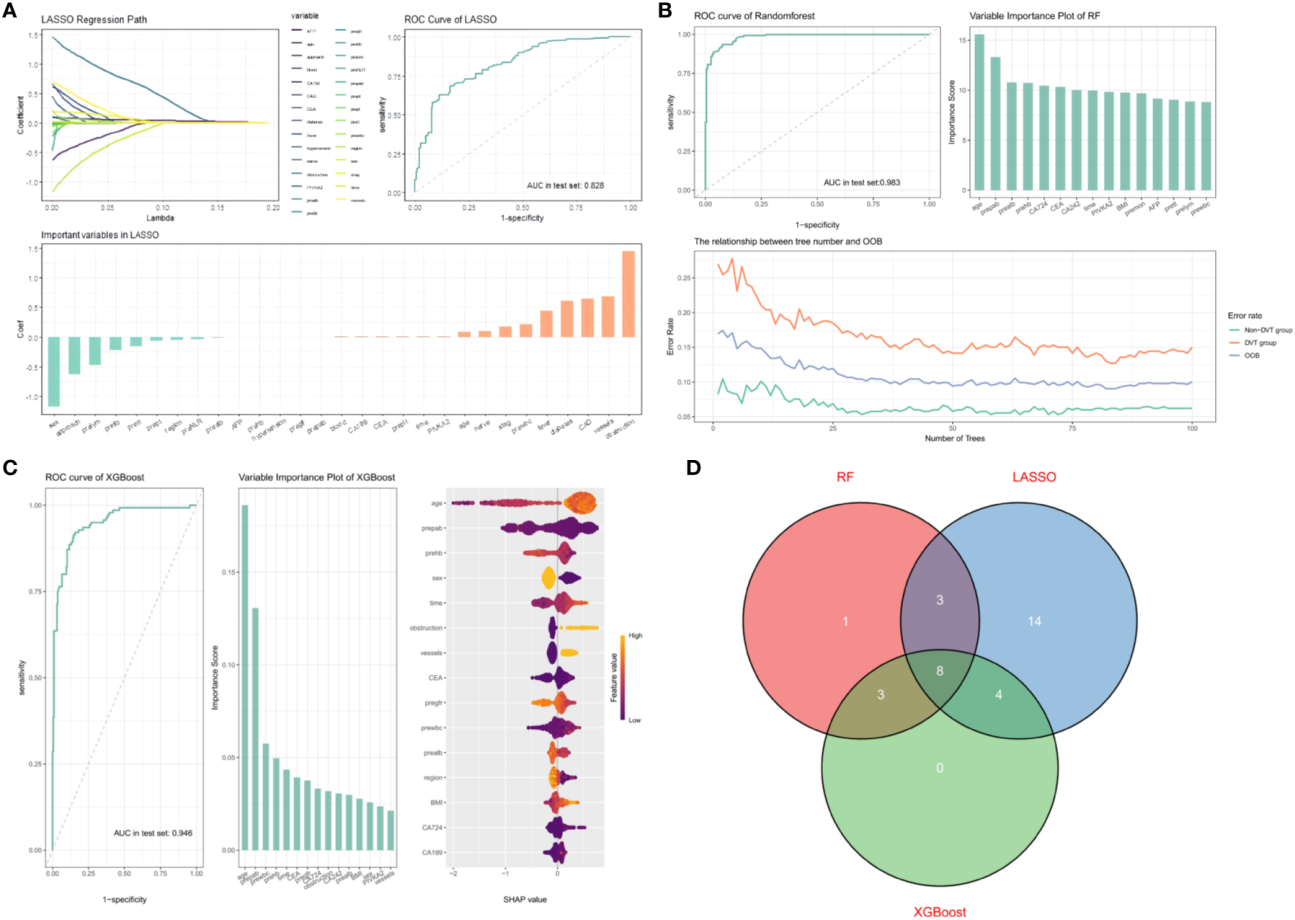
**(A)** AUC curve, path diagram, and importance ranking of selected feature variables from univariate combined with LASSO regression. 1. Penalization process of variables in LASSO. 2. Evaluation of predictive performance of LASSO model in testing set. 3. Feature importance ranking in LASSO model. **(B)** AUC curve, OOB plot, and importance ranking of selected feature variables from random forest. 1. Evaluation of predictive performance of RF model in testing set. 2.Feature importance ranking in RF model. 3. Relationship between number of trees and OOB (Out-of-Bag) error. **(C)** AUC curve, feature importance ranking, and SHAP visualization for XGBOOST model evaluation. 1. Evaluation of predictive performance of XGBOOST model in testing set. 2.Feature importance ranking in XGBOOST model. 3.SHAP value visualization for XGBOOST variables. **(D)** Eight common feature variables selected by three predictive models.

A total of 1134 patients with colorectal cancer receiving surgical treatment were involved after data imbalance. Patients were split into a training group with 796 cases and a test group with 338 cases in a 7:3 ratio. LASSO regression, as a shrinkage estimation method, achieves variable selection and complexity adjustment by formulating an optimization objective function with a penalty term. This study utilized LASSO regression to identify features including age, surgical procedure, acute intestinal obstruction, nerve invasion, preoperative lymphocyte count, preoperative fibrinogen, preoperative prothrombin time, coronary artery disease, and diabetes (**Figure 2A**). Random forest builds multiple decision trees through the random selection of data subsets and features. Each feature’s importance score reflects its contribution to the model’s predictions, allowing the extraction of the most predictive features and the identification of characteristic factors. Features including age, preoperative prealbumin, preoperative albumin, preoperative hemoglobin, CA724, CEA, and CA242 were selected (**Figure 2B**). Xgboost improves prediction performance by constructing multiple weak learners and using an additive model approach. The importance of features is assessed by calculating gain, coverage, and frequency for each one, identifying factors like age, preoperative prealbumin, preoperative white blood cell count, preoperative hemoglobin, preoperative glomerular filtration rate, BMI, and preoperative prothrombin time (**Figure 2C**). By comparing the selection results of LASSO regression, Xgboost algorithm, and random forest algorithm, we identified the common subset of features selected by these three methods. These selected features were eventually used to construct the model, including age, preoperative prealbumin, preoperative albumin, preoperative hemoglobin, operation time, PIKVA2, CEA, and preoperative neutrophil count (**Figure 2D**).

### 3.3 Model performance

**Figure 3:**
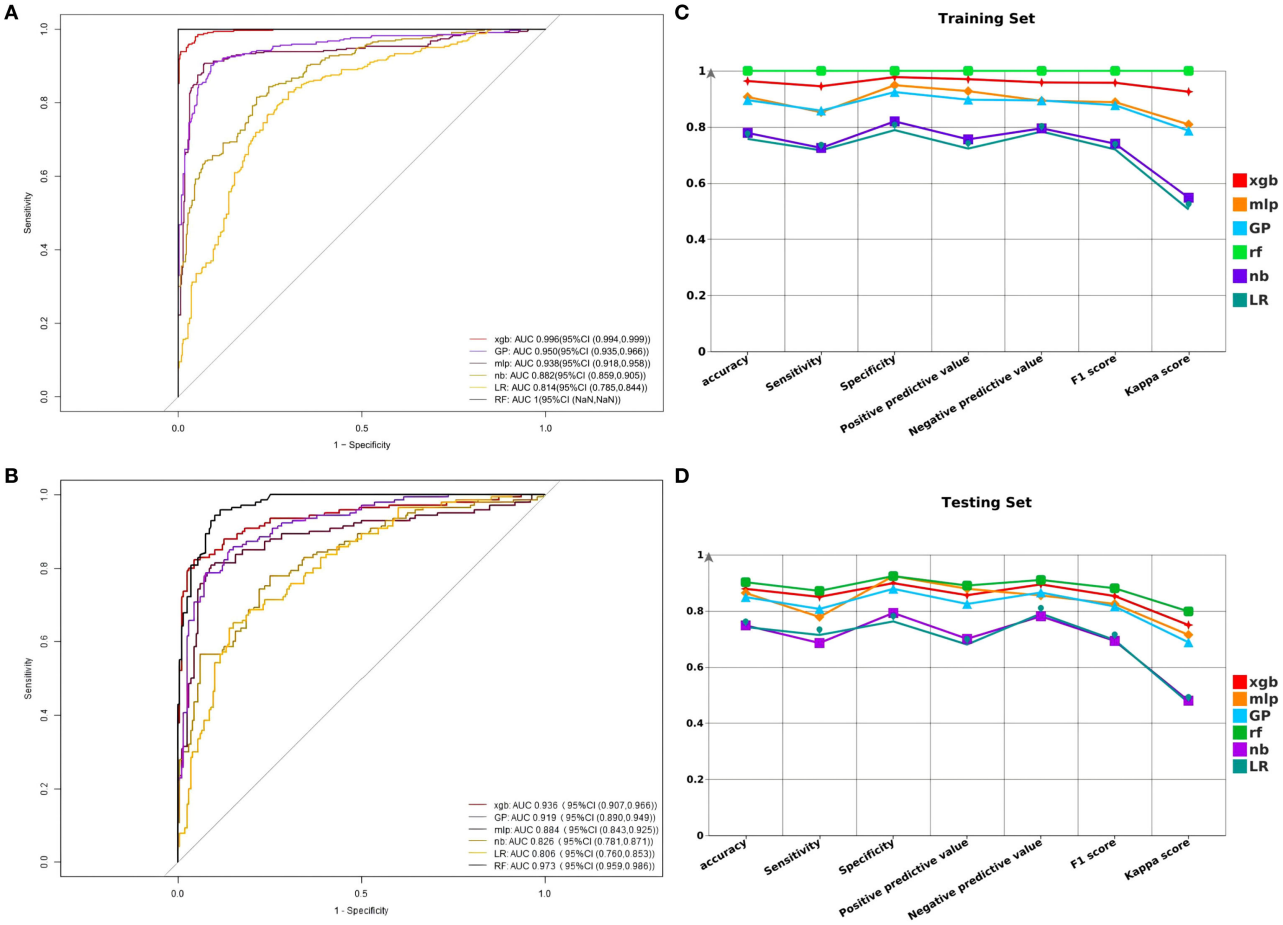
**(A)** Comparison of AUC models in the training set. **(B)** Comparison of AUC models in the testing set. **(C)** Comparison of F1 score, accuracy, sensitivity, specificity, positive predictive value, negative predictive value, and kappa value in the training set. **(D)** Comparison of F1 score, accuracy, sensitivity, specificity, positive predictive value, negative predictive value, and kappa value in the testing set.

In the training dataset, the RF model demonstrated excellent predictive performance with an AUC of 1.00, indicating very high prediction accuracy. In comparison, the AUC values for the remaining five models are as follows: XGB’s AUC is 0.996 (95% CI [0.994, 0.999]), GP’s AUC is 0.950 (95% CI [0.935, 0.966]), MLP’s AUC is 0.938 (95% CI [0.918, 0.958]), NB’s AUC is 0.882 (95% CI [0.859, 0.905]), and LR’s AUC is 0.814 (95% CI [0.785, 0.844]) (**Figure 3A**). The F1 scores of these models are as follows: RF 1.0, XGB 0.976, GP 0.878, MLP 0.889, NB 0.740, LR 0.720. In the testing dataset, the AUC values for XGB, GP, MLP, NB, LR, and RF are 0.936 (95% CI [0.907, 0.966]), 0.919 (95% CI [0.890, 0.949]), 0.884 (95% CI [0.843, 0.925]), 0.826 (95% CI [0.781, 0.871]), 0.806 (95% CI [0.760, 0.853]), and 0.973 (95% CI [0.959, 0.986]), respectively (**Figure 3B**). The F1 scores for XGB, GP, MLP, NB, LR, and RF are respectively 0.853, 0.816, 0.825, 0.693, 0.696, and 0.881. In this research, the accuracy, sensitivity, specificity, positive predictive value, negative predictive value, and kappa value of each model were computed and compared (**Figures 3C, D**). The RF model performed excellently in the training dataset. Due to concerns about potential overfitting, the XGB model was ultimately selected as the optimal model.

### 3.4 Model performance evaluation

**Figure 4:**
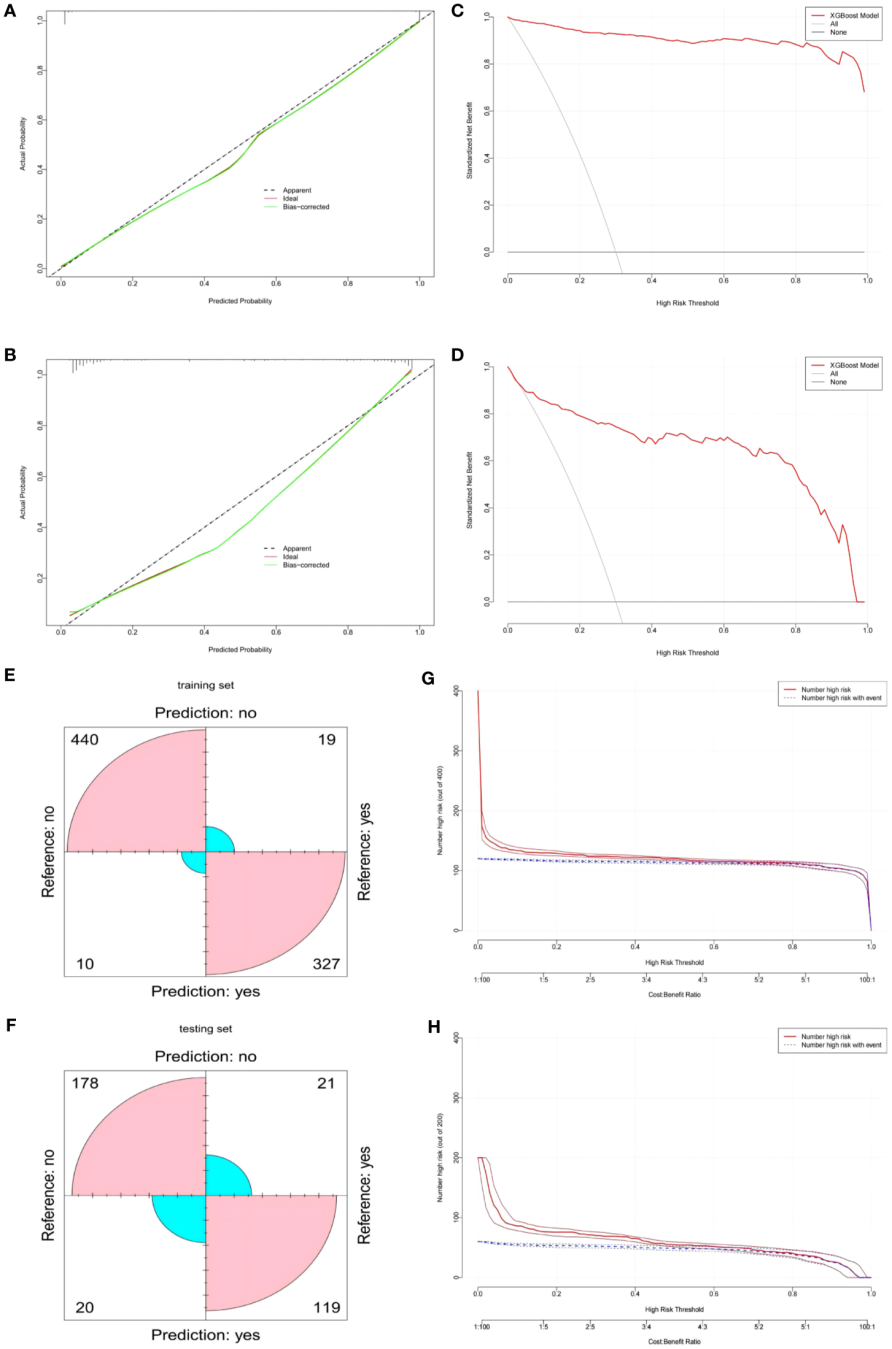
**(A)** XGBOOST model calibration curve in the training set. **(B)** XGBOOST model calibration curve in the testing set. **(C)** XGBOOST model clinical decision curve in the training set. **(D)** XGBOOST model clinical decision curve in the testing set. **(E)** XGBOOST model confusion matrix in the training set. **(F)** XGBOOST model confusion matrix in the testing set. **(G)** XGBOOST Model Clinical Impact Curve (CIC) in the training set. **(H)** XGBOOST model Clinical Impact Curve (CIC) in the testing set.

In our study, we evaluated the predictive accuracy and calibration of the model by analyzing calibration curves for the training and test sets. The calibration curve results showed that the model in the training set had high predictive accuracy, with a Somers’ D coefficient of 0.992 and an area under the ROC curve of 0.996, indicating good discriminatory power (**Figure 4A**). Additionally, the regression calibration slope of the training set model is 0.9934, close to the ideal value of 1.000, and the intercept is -0.0175, demonstrating excellent calibration ability. The Brier score is 0.038, reflecting the high reliability of the model’s predictions. In contrast, the model’s discriminatory power in the test set decreased but still maintained a high level, with an area under the ROC curve of 0.936 and a Somers’ D coefficient of 0.873 (**Figure 4B**). Decision curves for the training set (**Figure 4C**) indicate that the model’s net benefit is significantly above the baseline strategy. On the test set (**Figure 4D**), the model likewise exhibits good net benefit, particularly in the threshold probability range of 0.1 to 0.95, where it maintains a high level of net benefit. The confusion matrix results show the performance differences of the model across different datasets. In the training set (**Figure 4E**), the model correctly identified 440 true negatives and 327 true positives, with 10 false positives and 19 false negatives, the true positive rate is 85.0%, and the true negative rate is 89.7%.In the test set (**Figure 4F**), the model correctly identified 119 true negatives and 178 true positives, misidentifying 20 false positives and 21 false negatives, with a true positive rate of 85.0% and a true negative rate of 89.7%. During the model development process, we considered applying a penalty to the confusion matrix to reduce Type II errors (false negatives). Specifically, we explored methods such as adjusting the classification threshold and using weighted loss functions to impose a higher penalty on false negatives during model training. However, after several experiments, we found that while these adjustments could reduce false negatives, they also led to an increase in false positives, which in turn affected the overall performance metrics of the model (such as AUC and accuracy). Therefore, we ultimately decided not to apply such penalties to maintain the overall balanced performance of the model. Finally, we plotted Clinical Impact Curves (CICs) to evaluate the net benefit of the model with the highest diagnostic value in terms of clinical utility and applicability. Clinical Impact Curves (**Figures 4G, H**) offer insights into the model’s capability to predict high-risk patients at various cost-benefit ratio thresholds. The test set’s curve indicates that when prediction score probabilities exceed 65%, the model’s predictions for postoperative colorectal cancer patients align closely with those who actually develop lower extremity deep vein thrombosis, confirming the model’s high clinical efficacy.

### 3.5 Model-based interpretability analysis

**Figure 5:**
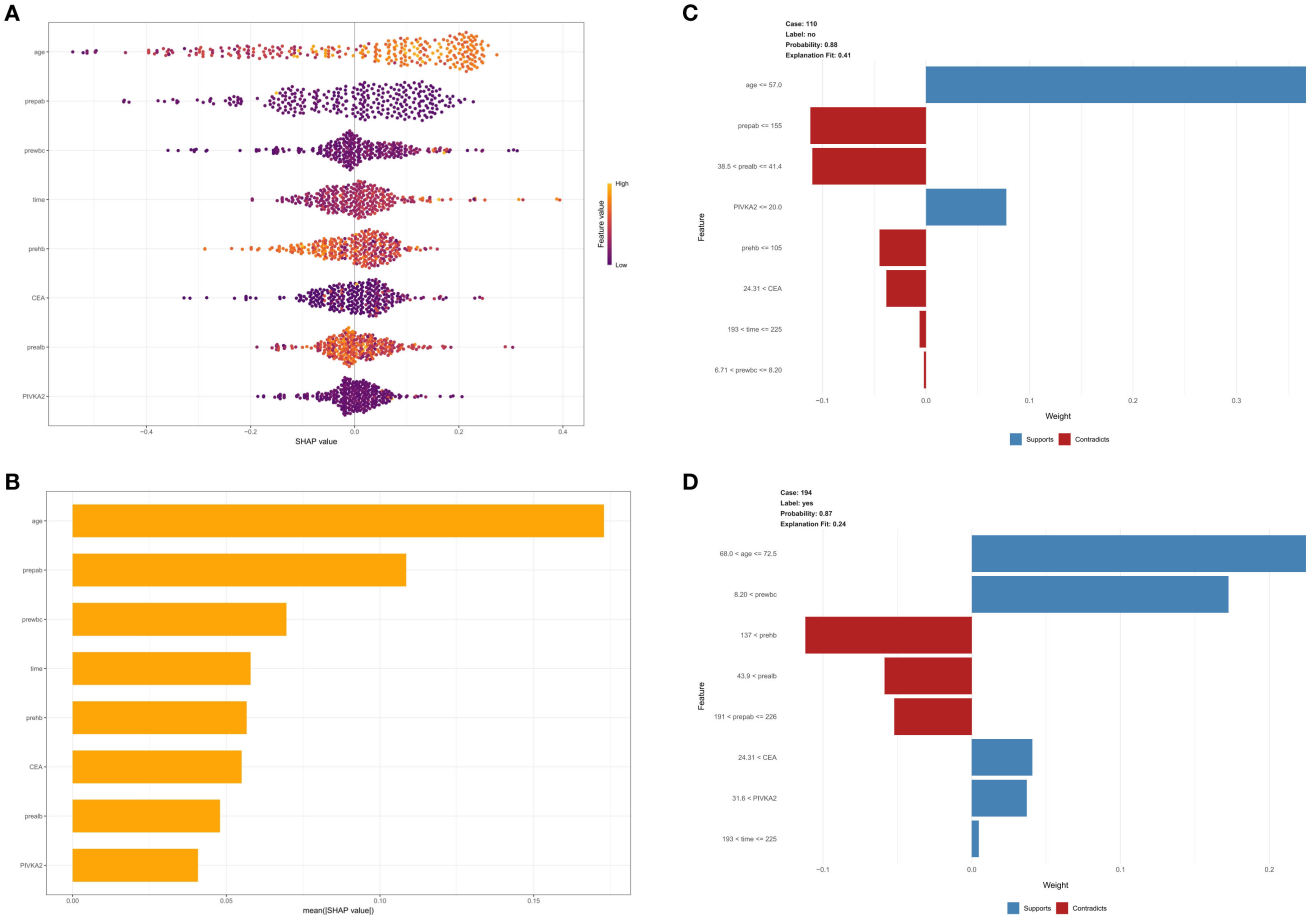
**(A)** SHAP interpretability analysis. The color gradient from purple to yellow represents the magnitude of the sample feature values. The vertical axis displays the importance ranking of features, along with the correlation and distribution of feature values with SHAP values. **(B)** Hierarchical importance ranking of features in the XGBOOST model. **(C, D)** Detailed interpretability analysis of two representative samples using LIME.

This study evaluated the relative importance of various factors affecting the susceptibility of colorectal cancer patients to developing lower extremity deep vein thrombosis post-surgery. **Figure 5A** visually represents this ranking, with each point indicating a sample and the color gradient from purple to yellow indicates the magnitude of sample feature values. The vertical axis shows the importance ranking of features alongside the correlation and distribution of feature values with SHAP values. **Figure 5B** illustrates the hierarchical significance of features in the XGB model. The vertical axis shows individual features ranked in descending order of importance, and the horizontal axis represents the average SHAP values. The analysis shows that age, preoperative albumin, preoperative white blood cell count, surgery duration, and preoperative hemoglobin are the top five ranked features in terms of importance, indicating their critical impact on the occurrence of DVT. To better understand the model’s decision-making process at the individual level, we performed detailed interpretability analyses using LIME on two representative samples(As illustrated in **Figures 5C, D**). Through model visualization, we can discern the impact of each feature on the model predictions for these specific instances.

## 4 Discussion

The migration of deep vein thrombosis from the lower extremities into the pulmonary artery through the circulatory system is a major trigger for fatal pulmonary embolism (10). The differences in disease onset and progression characteristics across various specialties result in varying incidence rates of lower extremity DVT (11). Literature reports indicate that the incidence of lower extremity deep vein thrombosis in colorectal cancer patients post-surgery is 40% (2). At present, there is a lack of effective evidence-based research on the risk factors, clinical characteristics, and targeted prevention and treatment measures for lower extremity DVT following gastrointestinal surgery. The American College of Chest Physicians Guidelines define cancer surgery as a high-risk factor for venous thromboembolism and recommend the use of intermittent pneumatic compression and certain medications (such as low molecular weight heparin, low-dose unfractionated heparin, and Xa inhibitors) to prevent the occurrence of venous thromboembolism (7). Caprini, Geneva, and Rapt scores are commonly used tools for assessing DVT, but they are limited in their applicability to colorectal cancer patients. The Caprini assessment rates all colorectal cancer patients undergoing abdominal surgery as high-risk, therefore, current risk assessment models are insufficient to identify patients truly at risk of DVT post-surgery. Many studies have examined the risk factors for postoperative DVT in colorectal cancer patients, such as open surgery, age, D-dimer, pulmonary disease, hemoglobin, and more (12, 13). Although many risk factors have been identified, the available assessment systems are still limited and unable to accurately predict the occurrence of postoperative DVT.

With the continuous advancement of surgical techniques for colorectal cancer, the differences in intraoperative factors are becoming less apparent. Therefore, we aim to develop a preoperative risk assessment tool similar to the Caprini score to facilitate early diagnosis and prevention of postoperative DVT in colorectal cancer patients.

Traditional approaches to identifying risk factors usually depend on developing risk models through univariate or multivariate regression, yet these models often ignore the interactions among variables and nonlinear relationships. In contrast, machine learning models are flexible enough to handle nonlinear and complex data structures, and can effectively address the challenges of high dimensional data and missing values. By training models on large datasets and continuously optimizing their performance, they improve prediction and classification accuracy (14–18). The SHAP algorithm utilizes the Shapley value concept from game theory, calculating the average contribution of each feature to the prediction. This approach enables us to thoroughly quantify each feature’s influence on the model’s overall predictions, thus providing a deeper understanding of the model’s workings (19). On the other hand, the LIME algorithm provides localized and transparent explanations by analyzing the feature importance of individual predictions. This local interpretability allows us to understand the reasons behind specific predictions in detail (20). The combination of these two approaches provides us a multidimensional model interpretation framework, capable of capturing global feature impacts and providing thorough insights into specific predictions.

In this study, we first used three machine learning models to construct a prediction model for DVT in patients with gastrointestinal tumors among postoperative colorectal cancer patients. Lasso, Xgboost, and Random Forest each filtered out 29, 15, and 15 feature vectors, respectively. In the end, we selected 8 common feature variables among the three models. During the feature selection process, we adopted a model-based feature selection method. This approach selects the most relevant features by evaluating each feature’s contribution to the model’s performance. Specifically, we employed algorithms such as Lasso regression, Xgboost, and Random Forest, which effectively handle high-dimensional data and identify features that most significantly impact the prediction results. Existing studies have shown that feature selection plays an important role in cancer prediction models; for example, Sun Tao employed LASSO regression combined with the Boruta algorithm for feature selection, thereby enhancing the accuracy of predicting the risk of pulmonary infection in lung cancer patients post-chemotherapy (21). The ROC curve constructed from these feature vectors indicates that the AUC values for Xgboost and Decision Tree are both greater than 0.900, and the AUC value for Lasso regression is 0.823. The findings indicate that the Lasso, Xgboost, and Decision Tree models have high clinical value in predicting postoperative DVT occurrence in colorectal cancer patients. In contrast, in the research conducted by Xiuying L et al. (22) the DVT model developed through the Caprini Risk Assessment Model exhibited an AUC value of merely 0.701, with a sensitivity of 80.6% and specificity of 56.3%. These comparative results highlight the superiority of the machine learning models in this study, providing powerful tools for accurately predicting postoperative DVT in colorectal cancer patients, indicating that machine learning technology has high potential for application in clinical research. We utilized six machine learning models to build and compare prediction models, from which we selected the optimal model. Through comparison, we found that the XGBOOST model has extremely high prediction accuracy, with an area under the ROC curve larger than 0.99. Additionally, the internally validated DCA and calibration curve confirmed the model’s consistency in net clinical benefit and prediction probability, indicating its high predictive value. Literature has shown that the Xgboost model has a higher predictive value for DVT prediction in gastrointestinal tumors, with an AUC value significantly higher than that of nomograms (23). Additionally, RuifengD et al. (24) constructed a model using the Xgboost model to predict early postoperative DVT in patients after hip surgery. In their study, the Xgboost model achieved an AUC of 0.991 ± 0.012 in the training cohort and an AUC of 0.982 in the validation cohort, with a sensitivity of 0.913 and a specificity of 0.998.The calibration and DCA curves in the validation cohort indicated good performance by the Xgboost model. Our study showed similar performance on these evaluation metrics, validating the model’s effectiveness and reliability.

Consistent with some studies (25), advanced age is a predictive factor for VTE occurrence. In our predictive model, SHAP feature importance ranking shows that advanced age is the most important predictive factor. This indicates that age plays a crucial role in predicting the risk of VTE occurrence. As age increases, reduced vascular elasticity and changes in coagulation mechanisms can increase the risk of thrombosis. Additionally, reduced activity and the presence of multiple comorbidities in the elderly also increase the likelihood of VTE occurrence.

Prealbumin is a protein synthesized in the liver, commonly used to assess nutritional status and liver function. Its levels can reflect a person’s nutritional state and inflammatory response (26, 27). Low levels of prealbumin are often associated with malnutrition, which may increase the risk of DVT (28). Malnutrition can lead to increased blood viscosity and endothelial dysfunction, thereby promoting thrombosis. Meanwhile, prealbumin levels decrease during acute inflammation or infection. The inflammatory response is a crucial mechanism in thrombosis as it can lead to endothelial damage and activation of coagulation factors (29, 30).

Studies have shown that there is a complex relationship between leukocyte activity and venous thrombosis, and the activity of inflammatory cells may play an important role in the natural history of thrombosis (31). Furthermore, research points out that when hematocrit is controlled, an increased white blood cell count (>12) is significantly correlated with the risk of thrombotic events (32). These discoveries highlight the significance of including white blood cell count as a factor in managing VTE, particularly among high-risk groups like surgical and cancer patients.

Our diagnostic tools encompass several additional features, including preoperative hemoglobin, preoperative albumin, CEA, and PIKVA2, all of which are essential preoperative laboratory checks. Additionally, we included surgery duration as a history-related feature. Some features in the tool have SHAP values that are inconsistent with clinical knowledge. However, it is important to consider that these features contribute differently to the overall model and should be viewed as a whole.

Our study has some limitations. Due to the limitations of retrospective studies, we were unable to include some highly valuable data that could be crucial and closely related to colorectal cancer. Despite extensive literature indicating that DD values might be closely linked to the occurrence of postoperative DVT (6, 33), unfortunately, due to a large number of missing values in preoperative DD, it was removed during preprocessing. We anticipate that with the advancements in genetics and bioinformatics, more predictive biomarkers will be identified and utilized, such as tumor genomic features in the Tic-ONCO model (34), among others. Additionally, due to limitations of the constraints of the data system, we could not perform extended observations on patients who were moved to rehabilitation facilities approximately 10 days after surgery. Finally, due to the lack of external validation, it is unclear whether our results are applicable to other populations, necessitating further research on more groups. In summary, these limitations hinder the clinical application of this predictive model, requiring further prospective studies with larger samples and meticulous design. As an initial exploration of this research theme, we hope this study offers some guidance for future prospective research.

## Data Availability

The original contributions presented in the study are included in the article/supplementary material. Further inquiries can be directed to the corresponding author.
